# Predicting the current potential and future world wide distribution of the onion maggot, *Delia antiqua* using maximum entropy ecological niche modeling

**DOI:** 10.1371/journal.pone.0171190

**Published:** 2017-02-03

**Authors:** Shuoying Ning, Jiufeng Wei, Jinian Feng

**Affiliations:** 1 Key Laboratory of Plant Protection Resources and Pest Management, Ministry of Education, Entomological Museum, College of Plant Protection, Northwest A&F University, Yangling, Shaanxi, P. R. China; 2 College of Agriculture, Shanxi Agricultural University, Taigu, Shanxi, P. R. China; 3 State Key Laboratory of Crop Stress Biology for Arid Areas, Northwest A&F University, Yangling, Shaanxi, P. R. China; University of Porto, PORTUGAL

## Abstract

Climate change will markedly impact biology, population ecology, and spatial distribution patterns of insect pests because of the influence of future greenhouse effects on insect development and population dynamics. Onion maggot, *Delia antiqua*, larvae are subterranean pests with limited mobility, that directly feed on bulbs of *Allium* sp. and render them completely unmarketable. Modeling the spatial distribution of such a widespread and damaging pest is crucial not only to identify current potentially suitable climactic areas but also to predict where the pest is likely to spread in the future so that appropriate monitoring and management programs can be developed. In this study, Maximum Entropy Niche Modeling was used to estimate the current potential distribution of *D*. *antiqua* and to predict the future distribution of this species in 2030, 2050, 2070 and 2080 by using emission scenario (A2) with 7 climate variables. The results of this study show that currently highly suitable habitats for *D*.*antiqua* occur throughout most of East Asia, some regions of North America, Western Europe, and Western Asian countries near the Caspian sea and Black Sea. In the future, we predict an even broader distribution of this pest spread more extensively throughout Asia, North America and Europe, particularly in most of European countries, Central regions of United States and much of East Asia. Our present day and future predictions can enhance strategic planning of agricultural organizations by identifying regions that will need to develop Integrated Pest Management programs to manage the onion maggot. The distribution forecasts will also help governments to optimize economic investments in management programs for this pest by identifying regions that are or will become less suitable for current and future infestations.

## Introduction

Climate is one of the principal factors defining the potential range of insects and climate change directly affects the distribution of species [1–2]. Considerable evidence suggests that the average global temperatures will increase by 2–4°C between the present and 2100 under several different greenhouse gas emission scenarios [3–4].Global warming will likely affect almost all aspects of insect life history and population dynamics such as development rate, voltinism, and distribution range [5–8]. Rising temperatures can significantly influence the key physiological characteristics that affect the distribution and seasonal activity of insect pests [9–10] and change the likelihood of severe pest damage by turning climatically unsuitable habitats into suitable ones or vice versa [11–12]. Because of the sensitivity of insects to weather conditions, global climate change will potentially drastically alter pest outbreaks [13–15].

The onion maggot, *Delia antiqua*, is a worldwide and serious chronic pest with a narrow host range within the genus *Allium* such as Onion (*Allium cepa* L.), Scallion (*Allium fistulosum* L.) and Garlic (*Allium sativem* L.) that significantly damages plants in subtropical regions throughout the world [16–17]. The initial occurrence of onion maggot was reported in the USA in some regions of Wisconsin in the early twentieth century [18]. This pest continues to cause serious damage in Asia, particularly in China and Japan, Europe, and North America [19–21]. The onion maggot has three or four generations annually, and the first generation causes the greatest economic loss because the larvae kill young plants [22]. Second- and third-generation larvae cause little damage to the crop relative to the first generation, but feeding injury can distort bulbs and allow entry of pathogens, both of which render crops unmarketable [23–24].

Many studies have investigated the ecology and physiology of the onion maggot, and the current worldwide distribution of the pest is fairly well known. However, it is likely that the future distribution of this pest will be expanded because of the continued effects of global warming. Currently, no one has used distribution records and current and predicted climactic data to predict future changes in the distribution of this pest. Most of *Allium* plants have very high economic value and they also rank high position among vegetables production throughout the world [25]. This type of information will be absolutely necessary for scientists and farmers involved in production of *Allium* crops throughout the world to develop future monitoring and management strategies than can minimize losses, particularly in areas where the pest has not caused serious damage prior to global warming.

Several distribution models have been developed to provide information about the future potential effects of climate change on the future distribution of insect species. The most well known models to predict the distribution of insects based on climactic inputs include: GLM, GAM, BRT, BIOCLIM, CLIMEX, GARP and MaxEnt, etc [15,26]. In recent comparisons using several types of algorithms to predict species distribution, MaxEnt was regarded as the best-performing model using presence-only data and showed a better performance comparable to 16 other algorithms, such as general linear models (GLM) [27–28]. In several previous studies, MaxEnt was applied to predict the potential distribution of species, such as: Stink bug (*Halyomorphahalys*) [29], Winter annual grass (*Bromustectorum*L.) [30], and Fruit fly (*Bactroceradorsalis*) [31], and Spiny pocket mice (*Rodentia*: *Heteromyidae*) [32], etc.

In this study, we collected the occurrence data for onion maggot based on extensive references and we used MaxEnt to estimate the current potential distribution of *D*. *antiqua* and to predict the future distribution of this species in 2030, 2050, 2070 and 2080.Based on the results of these predictions, we provide a theoretical reference framework for the management and prevention of onion maggot.

## Methods

### Occurrence data and study area

Occurrence data were collated from surveys published in the research literature. A total of 128occurrence records for *Delia antiqua* were used in our analysis. Geo-coordinates for each chosen datapoint were either referenced from information presented in the literature or by using Google Earth coordinates (S1 Table).

Occurrence data are often biased due to differences in sampling intensity. In order to reduce the impact for clustered occurrence data on MaxEnt models, we used a raster (grid size approximately 10km^2^) and randomly selected one record per cell in this study [33–34]. This method reduced the number of occurrences to 99 points used for further study.

Some studies using environmental variables over large areas (eg. all over the world) have led to conservative predictions of current species distribution and under-estimated the extent of climate change [33–34]. Moreover, some studies suggested that the onion maggot is a chronic pest of cultivated *Allium* sp. throughout the Holarctic region [16,35]. Therefore, we selected North America, Europe and West Asia, East-Asia and Japan respectively as the target areas in our modeling.

### Environmental layers

#### Current environmental parameters

Nineteen environmental variables which had 30’ spatial resolution (~1km) were used in our present study (Bio1-19) (http://www.worldclim.org/) [26,36]. Correlation analyses were used to select bioclimatic layers. We selected layers that were not highly correlated (r<0.9) because many studies have shown that highly correlated variables affect the results for species distribution modeling (S2 Table) [37–40]. Furthermore, many previous studies of the biology and persistence of onion maggot infestations show that temperature is a basic impact factor for subterranean pests, such as *Delia antiqua*, because it determines the extent of spread and induces specific physiological behaviors [18–20, 22]. Temperature not only determines the suitability of habitats for this pest, but also induces key physiological processes such as diapause [41–42]. The seven bioclimatic layers included in our modeling studies were obtained from Worldclim (Table 1) [36].

**Table 1:** Factors included as predictor variables to model the potential global distributions of *Delia antiqua*.

Environmental Variables	
Bio01	Annual mean temperature (°C)
Bio02	Mean diurnal temperature range (mean(period max-min)) (°C)
Bio04	Temperature seasonality (C of V)
Bio05	Max temperature of warmest month (°C)
Bio06	Min temperature of coldest month (°C)
Bio13	Precipitation of wettest month (mm)
Bio14	Precipitation of driest month (mm)

#### Future environmental parameters

For future climatic predictions, we used downscaled predictions available in the Worldclim database from over 24 different GCMs used in the IPCC Fourth Assessment Report [4]. The IPCC SRES (Intergovernmental Panel on Climate Change Special report of Emission scenarios) included two emissions scenarios, the A2 and B2 scenarios. To illustrate the worst-case climate change prediction, we chose A2 as the future climate scenario. The A2 emissions scenario assumes relatively rapid population growth and relatively high global CO_2_ emissions increasing to almost 5 times the1990 values by 2100. The B2 scenario predicts slower continuing population growth and diverse technological changes resulting in global CO_2_ emissions just double their 1990 values by 2100. These scenarios covered 7 different 30 year running mean periods: 2010–2039 (2020s), 2020–2049 (2030s), 2030–2059 (2040s), 2040–2069 (2050s), 2050–2079 (2060s), 2060–2089 (2070s) and 2070–2099 (2080s). In this study,4 mean periods were used for future prediction: 2030, 2050, 2070 and 2080. All dimensions were set at 30’ spatial resolution for further analysis.

### Ecological niche modeling

Ecological niches and associated potential geographic ranges can be approximated using correlative algorithms that relate known point-occurrence data to digital GIS data layers, and summarize spatial variations in these layers in multidimensional environmental space [43].

The maximum entropy method as implemented in MaxEnt version 3.3.3 software is used to create models of the suitable current and future niche distributions of species [44–47] and has been widely contributed to estimate species distributions [48–49]. This software was used in our study because of three reasons: 1) it has been better than other algorithms in predicting species’ distributions that reflect the true physiological or climatic conditions [50]; 2) it performs better than other models such as BIOCLIM and GARP in predicting the effects of climate change on distribution [27,51]; 3) it finds the probability distribution of maximum entropy (that which is closest to uniform) subject to constraints imposed by the observed spatial distributions of the species and environmental conditions [45].

In this study, the algorithm was run for the variables at current climatic conditions with 5 replicates, 500 iterations and 10000 random background points. Then, we performed modeling projections for future climate scenarios (2030, 2050, 2070, and 2080). We let MaxEnt select both suitable regularization values and functions of climate variables automatically, which it achieves based on considerations of sample size. MaxEnt outputs a continuous index, ranging from 0 to 1, that is an indicator of relative suitability for the species, based on the principle of maximum entropy, as constrained by the input occurrence data. We ran the models using the default regularization values that have been tuned to perform well across a variety of organisms and regions [52].

### Model evaluation

To assess the performance of the models, the areas under the curve (AUC) metrics of the receiver operator characteristics (ROC) curves were used in the current study [53]. The AUC was obtained by the threshold independent receiver operating characteristic (ROC) analysis [49]. A ROC curve shows the performance of a model whose output depends on a threshold parameter, it tests whether a model classifies species presence more accurately than random predictions. A perfect model has an AUC value of 1, but performance is excellent when the AUC value is > 0.9 [54]. In the process of modeling, 80% of occurrence localities were randomly selected as training data, while the remaining 20% served for testing the resulting models [55].

To improve displays of predictions in this study, the logistic output of MaxEnt, which ranges from 0 (unsuitability) to 1 (high suitability), was used [52]. Conversion of the continuous suitability index maps to binary habitat and non-habitat charts required a probability threshold to determine the potential change in current and future habitats of species. To define habitats and non-habitats for *Delia antiqua*, the “maximum training sensitivity plus specificity” threshold was employed in our study [56].

The predicted results were classified into four levels of suitability: unsuitable(0-threshold), marginally suitable (threshold-0.4), moderately suitable (0.4–0.6) and highly suitable areas (0.6–1).

We used Arcview 3.3 (ESRI) to output our map in our studies.

## Results

### Statistical model evaluation

Based on known occurrences of onion maggot and current climate data, we generated geographic distribution maps predicting areas where onion maggot might occur. The model performance for this pest was better than random (AUC = 0.94 for training data and AUC = 0.923 for test data); thus, the model produced highly effective in predicting the suitable habitat area for this species. Our “maximum training sensitivity plus specificity” threshold value of 0.29 was obtained from the 5 time replication.

### Current and potential distribution

**Fig 1:**
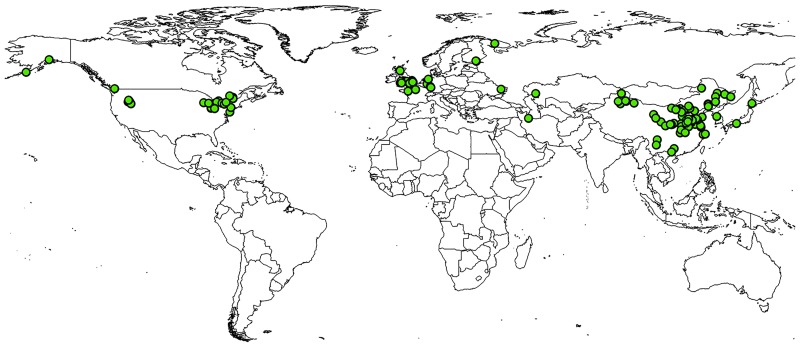
The current global distribution of onion maggot, *Delia antiqua*. Reprinted from ESRI data under a CC BY license, with permission from Environmental Systems Research Institute, Inc., original copyright 2016.

**Fig 2:**
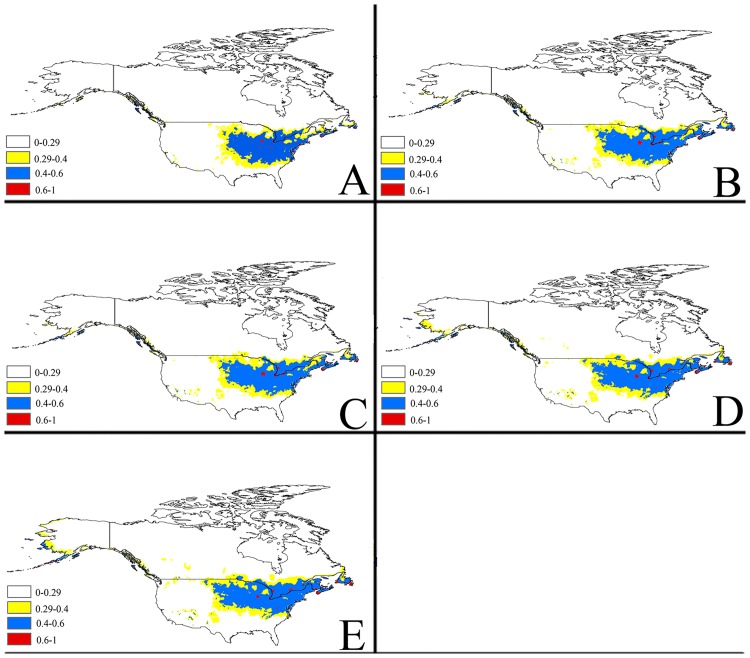
Future potential suitable habitats for *Delia antiqua* on the North America continent predicted by MaxEnt. The letters show predictions for the current, 2030, 2050, 2070and 2080 timeframes (A: Current; B: 2030; C: 2050; D: 2070; E: 2080). Red = highly suitable areas; Blue = Moderately suitable areas; Yellow = Marginally suitable areas.

**Fig 3:**
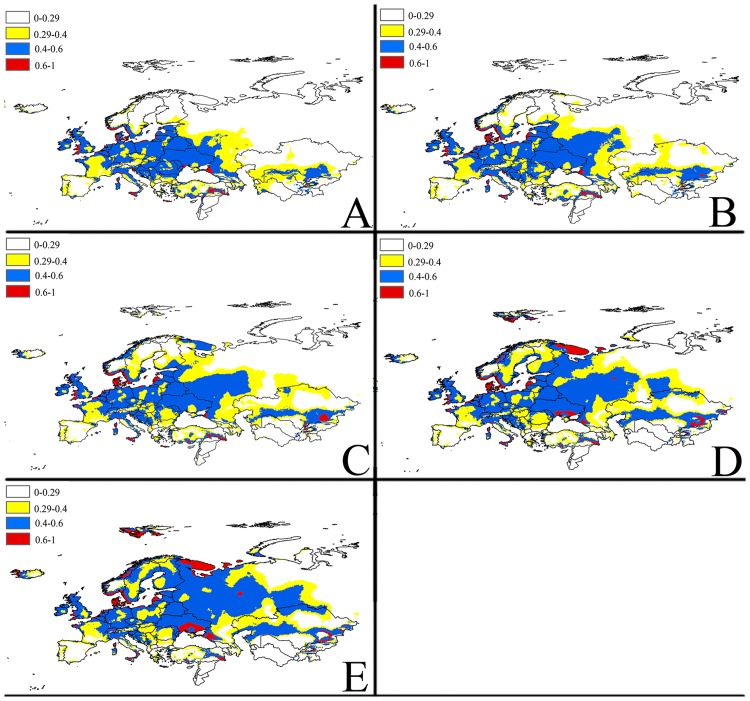
Future potential suitable habitats for *Delia antiqua* on Europe and West Asia continent predicted by MaxEnt. The letters show predictions for the current, 2030, 2050, 2070and 2080 timeframes (A: Current; B: 2030; C: 2050; D: 2070; E: 2080). Red = highly suitable areas; Blue = Moderately suitable areas; Yellow = Marginally suitable areas.

**Fig 4:**
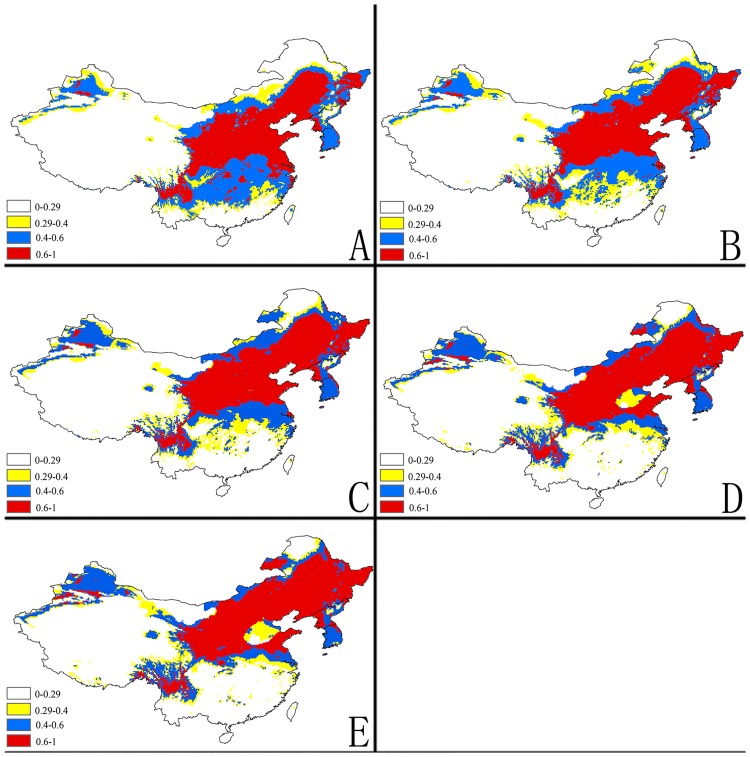
Future potential suitable habitats for *Delia antiqua* on China & the Korea continent using MaxEnt. The letters show predictions for the current, 2030, 2050, 2070and 2080 timeframes (A: Current; B: 2030; C: 2050; D: 2070; E: 2080). Red = highly suitable areas; Blue = Moderately suitable areas; Yellow = Marginally suitable areas.

**Fig 5:**
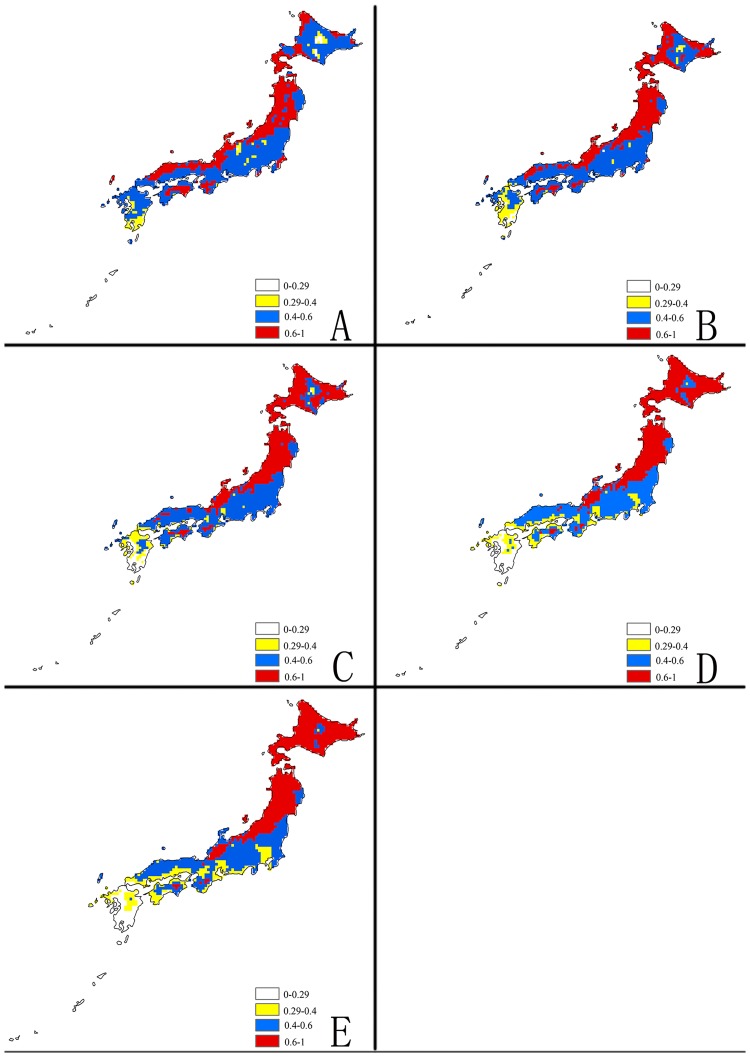
Future potential suitable habitat for *Delia antiqua* on the Japan continent using MaxEnt. The letters show predictions for the current, 2030, 2050, 2070and 2080 timeframes (A: Current; B: 2030; C: 2050; D: 2070; E: 2080). Red = highly suitable areas; Blue = Moderately suitable areas; Yellow = Marginally suitable areas.

The present distribution of *Delia antiqua* in the world according to the occurrence records we obtained from the literature is illustrated in Fig 1. The current climate predictions from MaxEnt showed the following potential distribution patterns for the various parts of the world: (1) wide areas of North America, particularly the Central and Eastern parts of the United States (especially the Atlantic ocean coastal areas, southern coastal regions of Alaska, and some Northern and central states) and the Eastern Southern areas of Canada bordering the US (2) Europe and western Asia, particularly the Eastern European and Western Asian countries near the Caspian sea and Black Sea (especially England, Ireland, Denmark, Ukraine, Turkey and Kazakhstan) (3) Eastern Asia- Most of China, Japan, North Korea and South Korea (Figs 2–5).

The model predictions for current distribution and the actual recorded distribution data shown in the occurrence records fit together very well. Seventy-nine percent of the occurrence records from around the world occurred in the areas that the model predicted would be highly suitable areas for onion maggot development. The model predictions for areas around the world that would be moderately or marginally suitable for onion maggot development are extensive, mainly including: North America, Central and Western Europe, Eastern Asia, and the Caspian and Black Sea regions. In the USA and Europe these predicted regions that are less optimal for development of this pest are actually much larger than the predicted highly suitable areas. In China, Japan and North Korea, there are very large regions predicted to be highly suitable, which are slightly less than the sum of moderately and marginally suitable regions. But in South Korea, almost all regions are predicted to be moderately suitable.

### Future climate predictions

The MaxEnt models with A2 emission scenarios for potential distribution of *Delia antiqua* for 2030, 2050, 2070, and 2080are illustrated in (Figs 2–5). To simplify the figures, the global distribution is subdivided into three regions: North America, Europe and West Asia, East Asia.

#### a) North America

In North America (Fig 2), the MaxEnt model currently predicts that a few regions in Northern and North-eastern coastal states of America, such as New York, New Jersey, Ohio, Maryland, Indiana, Illinois and Iowa, have highly suitable climate for *Delia antiqua*. Also, the model predicts that most of the Mid-northern and North-eastern coastal areas of America are moderately and marginally suitable climatic areas for this species. In the future predictions in 2030 to 2080, these less optimal suitable areas will gradually expand to include more of the Northern and Mid-western states such as Kansas, Oklahoma, Colorado and Utah. In addition, the model predicts that highly suitable areas for onion maggot in Southern Alaska will greatly increase between 2030 to 2080.

In Canada, the Southern areas of Ontario and Quebec just north of the US border will remain mostly moderately suitable for the pest in future climactic predictions. The unsuitable areas in Newfoundland and New Brunswick will gradually become marginally suitable for onion maggot by 2030 and this area will continuously increase or become even more suitable regions. In addition, the predictions showed that Southern Nova Scotia and Southern New Brunswick will be highly suitable for the pest by 2030, and then steadily increase (Fig 2).

**Fig 6:**
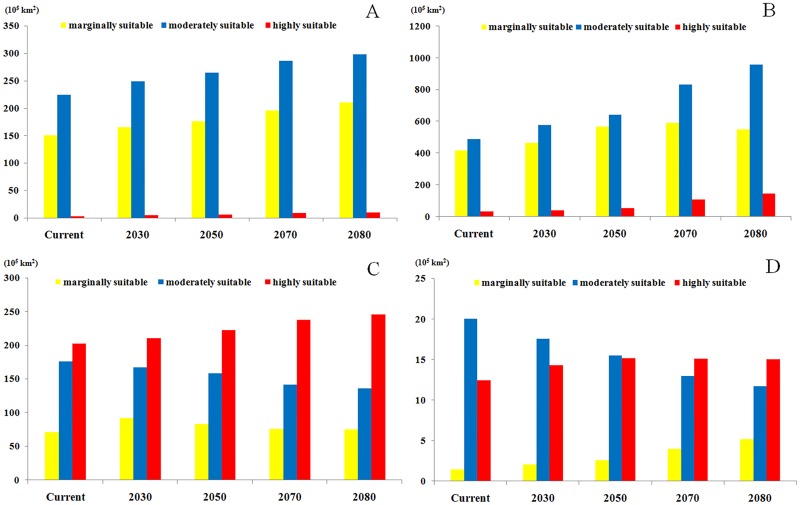
Comparison of future potential suitable habitat areas for *Delia antiqua* by MaxEnt for time frames on different continents: Current,2030, 2050, 2070 and 2080. Red = highly suitable areas; Blue = Moderately suitable areas; Yellow = Marginally suitable areas. A: North America; B: Europe and Asia; C: China & Korea; D: Japan.

In general, MaxEnt predicts that highly suitable areas for onion maggot in North America will gradually increase until 2080. Also, the moderately and marginally suitable areas will gradually increase by more than 39% and 32% in the future, respectively. The highly suitable areas will also increase and then will stabilize to 10.24 sq km by 2080 (Fig 6A).

#### b) Europe and West Asia

The MaxEnt model predicted that a few areas in North-Western Europe and the Western Asian regions near the Caspian sea and black Sea will be highly suitable for Onion maggot between 2030 to 2080, particularly in Southern England, Southern Norway, Southern Sweden, Netherlands, Belgium, South-western Russia, Southern Turkey and Southern Kazakhstan, etc. By 2080, the range of maximally suitable areas for this pest will spread to many regions in Northern Europe such as Iceland and North-western Russia. Also, the model predicts that moderately and marginally suitable areas for the onion maggot will greatly increase between 2030 to 2080. By 2080, the model predicts that most countries of Europe will have suitable living conditions for this pest, including large parts of Western Europe, Western Russia and Northern Kazakhstan.

In Europe and West Asia, highly, moderately and marginally suitable areas for onion maggot are predicted to continuously increase until 2080, and the area will stabilize to 146.35sq km, 960.2sq km and 550.65sq km, respectively (Fig 6B).

#### c) East Asia

In China, almost all of the Northeastern, Northcentral and Central regions and some of the Northwestern and Southeastern regions are currently highly suitable for the onion maggot. The MaxEnt model shows that the total area of maximum suitable land will slightly increase in the future although the major part of this area will shift from south to north and will include scattered distributions in many provinces. The suitable areas will decrease gradually in southern parts from 2030–2080 and Northern Xinjiang will become more suitable. In addition, most of Western and the Northern most Chinese regions will remain unsuitable for onion maggot outbreaks (Fig 4).

Currently, Northern Japan, Western North Korea, and a few regions in Northeast of South Korea are highly climatically suitable for *Delia antiqua* (Figs 4 and 5). The model predicts that the proportion and distribution of highly suitable areas will continuously increase in Korea and Northern Japan from 2030–2080. In contrast to the rise in suitability in northern regions, Southern Japan will become progressively less suitable in the future, and then will become mostly moderately or marginally suitable by 2080.

The predictions for highly and marginally suitable regions in China and Korea will increase 21% and 5%, respectively. In contrast, the moderately suitable areas will decrease 22% by 2080 (Fig 6C). The overall predictions for habitat suitability in Japan will remain fairly constant, except that many highly suitable regions will become only marginally suitable habitat (Fig 6D).

## Discussion

In this study, the MaxEnt model’s current predictions of the most suitable habitats for the development of the onion maggot generally agreed with available host occurrence records. Analyses showed that MaxEnt produced highly accurate predictions of AUC value that was greater than 0.9. Future model predictions from 2030–2080 derived from a IPCC climate change scenario showed that climatic changes would greatly affect the world-wide distribution of this pest, but the specific effects will vary among different locations. In general, the model predicted that there would be an expansion of highly and moderately suitable habitats in most areas in response to global warming, but these more suitable habitats will actually decrease in a few specific locations. Furthermore, the general locations of the most favorable habitats would shift substantially in most countries in response to the predicted climactic changes.

Previous research has demonstrated that the suitable temperature range for onion maggot development is 16–25°C and this species has unique physiological and biological characteristics such as diapause caused by temperature and given thermo-periodic eclosion rhythms under different photoperiods [41–42, 57].

Our present study showed that temperature will affect the distribution of onion maggot and verified the previous hypothesis that the main distribution zone is throughout the Holarctic region (35–60°N) [35]. The results indicated that regions with very hot summers or cold winters would not be suitable for *Delia antiqua*. Because of the greenhouse effect and rising global temperatures, the areas that are currently too cold for onion maggot development will become suitable habitats in the future, particularly in Newfoundland in Canada, Iceland, Norway, Sweden, North-western Russia. Also, global warming will cause currently unsuitable habitats in Northern Kazakhstan and Western Russia, to become suitable from current to 2080. This increase in habitat suitability in these regions then may facilitate the spread of this pest from Europe to Asia. In contrast, global warming will cause currently suitable habitats such as Southern China to become unsuitable because these areas will become too hot for onion maggot development in the future.

The future predictions of worldwide changes in the suitability of onion maggot habitats in response to global warming can be used by scientists and members of agricultural communities in making informed choices about planting *Allium* species in different locations. Former studies have focused on outbreak regularity, physiological features and pesticide control strategies in areas that are already heavily infested with this pest, but have not considered strategies to limit the spread of this pest to future areas rendered to be suitable for habitats for the pest because of global warming [18, 20].

The model developed in this study to predict shifts in onion maggot distribution patterns on a global scale, will be beneficial in developing monitoring strategies to detect future infestations in currently un-infested regions. Pest forecasting in early infestation stages and implementing subsequent preventative strategies has been widely accepted as one of the most promising and cost-effective ways in managing pests [16]. For example, in areas in North American and European countries that are predicted to become suitable habitats for onion maggot in the future, monitoring networks can be deployed for early detection of infestations and precautionary agricultural strategies to prevent outbreaks can be implemented. These preventative strategies could include: planting less susceptible varieties of *Allium*, rotating other crops annually into planting systems, and sanitation practices to limit availability of plants and bulbs in fields after harvest. Finally, after early detection of first generation larvae, it is important to focus on larvae eradication to prohibit further outbreaks of the species in the following generations in the highly suitable areas [58]. Previous studies have demonstrated the importance of changing planting time to control and minimize the risk of pest outbreaks. Delaying the planting time has been investigated as a novel management approach for *Delia antiqua* [59].

Although large parts of the world were forecasted to have suitable conditions for *Delia antiqua*, data from several regions, such as some European and Central Asian countries, is limited. Other biotic factors such as host-plant availability, cultivation practices, competition, and lack of dispersal opportunities could preclude this species from becoming a serious pest in these areas [59–62]. Therefore, in potentially suitable regions for future infestations, we suggest that special precautions should be taken to limit potential introductions by human activities such as cultivation and agriculture product importation. In addition, research programs should be developed to introduce or conserve natural enemies, and IPM programs should be implemented so that minimal amounts of pesticides are used that are relatively safe to natural enemies.

## Supporting information

S1 Table Occurrence data of *Delia antiqua*. (DOCX)

S2 Table Correlation analysis of environmental variables. (DOCX)
