# Supplementary material for: Predicting the current potential and future world wide distribution of the onion maggot, Delia antiqua using maximum entropy ecological niche modeling
Source: PLoS One. 2017 Feb 3;12(2):e0171190. doi: 10.1371/journal.pone.0171190 (PMC5291381; doi:10.1371/journal.pone.0171190)
Supplement: S1 Table — (DOCX) [file pone.0171190.s001.docx]

**Table S1:** The current global distribution of Onion maggot, *Delia antiqua*

| **Locations** | **Latitude** | **Longitude** | **Derivation** |
| --- | --- | --- | --- |
| Ada County, Idaho,  United States | 43.48 | -116.24 | Thomas M.M., 1993, Ovipositional Patterns and Larval Movement of *Delia antiqua* (Diptera: Anthomyiidae) on Sprouted Bulb and Seedling Onions, Journal of Economic Entomology, 86(5): 1440-1445 |
| Anchorage, Alaska,  United States | 61.17 | -149.80 | http://www.gbif.org/ |
| Anqiu city, Shandong, China | 36.17 | 119.04 | Anqiu county Agriculture Information network (www.aqny.gov.cn) |
| Anyang city, Henan, China | 36.10 | 114.38 | Agriculture Technology Formation,1979, The occurrence regularity of Onion fly in garlic field, Bulletin of Agricultural Science and Technology, 9: 36 |
| Atyrau, Kazakhstan | 47.09 | 51.88 | Kuo C.G., Ravza F.M. & Thomas J.K., 2005, Increasing Market-oriented Vegetable production in Central Asia and the Caucasus through Collaborative Research and Development, The world vegetable center, conference paper |
| Bianyuan town, Feicheng, Shandong, China | 36.01 | 116.88 | Chen jing, Fan hui-xia, 2011, Comprehensive control of disease and pest under leek field, Northwest Horticulture, 4:42-43 |
| Bradford West Gwillimbury, Ontario, Canada | 44.06 | -79.57 | Hoepting C.A., Scott-Dupree C.D., Harris C.R., Mcdonald M.R., 2004, Insecticide and Fungicide Combinations to Optimize Control of Onion Maggot (*Delia antiqua*) and Onion Smut (Urocystis cepulae) in Ontario, Journal of Vegetable crop production, 9(2):49-63 |
| Bürstadt, Hessen, Germany | 49.65 | 8.46 | Otto M., Hommes M., 2000, Development of a simulation model for the population dynamics of the onion fly *Delia antiqua* in Germany, EPPO conference on Warning Services for plant protection, 30:115-119 |
| Cangshan county,  Linyi, Shandong,  China | 34.86 | 118.07 | Zhang hong-cai, 1994, The occurrence regularity, damage, and prevetion of onion fly in garlic field, China Plant Protection, 5:4 |
| Canyon County, Idaho, United States | 43.65 | -116.73 | Thomas M.M., 1993, Ovipositional Patterns and Larval Movement of *Delia antiqua* (Diptera: Anthomyiidae) on Sprouted Bulb and Seedling Onions, Journal of Economic Entomology, 86(5):1440-1445 |
| Celeryville, Ohio,  United States | 41.02 | -82.73 | Erol Y., Casey W.H., 2003, Interaction between cyromazine and the entomopathogenic nematode Heterorhabditis bacteriophora Poinar "GPS11" for control of onion maggot, *Delia antiqua* (Meigen), Crop protection, 22:923-927 |
| Chabuer county,  Yili, Xinjiang,  China | 43.86 | 86.12 | Wei dong, Jia shuang-rui, 2009, Prevetion of main disease and pest on leek, Rural Science & Technology, 4:55-56 |
| Cheng county, Longnan,  Gansu, China | 33.74 | 105.73 | Tian hui-yao, Zhang pei-fang, Hou shu-yin, Li lin-yan, Yang yan-hong, 2007, Main diseases and pests of garlic and its comprehensive control technology in Longnan, Northwest Horticulture, 5:31-33 |
| Chicheng county,  Zhangjiakou,  Hebei, China | 40.92 | 115.81 | Xue zhen-yu, 1989, The determination of control strategy and period of Onion fly, Vegetables, 3:32 |
| Cloverdale, Surrey, British Columbia, Canada | 49.09 | -122.70 | Vernon R.S., Hall J.W., Judd G.J.R., Bartel D.L., 1989, Improved Monitoring Program for *Delia antiqua* (Diptera: Anthomyiidae), Journal of Economic Entomology, 82(1):251-258 |
| Colchester,  United Kingdom | 51.89 | 0.90 | http://www.gbif.org/ |
| Dafeng, Jiangsu,  China | 33.20 | 120.46 | Wang yong-shan, Chen hua, Wang feng-liang, 2003, The occurrence regularity and control measures of onion maggot in garlic field, Northern Jiangsu province, Journal of Changjiang Vegetables, 8:35 |
| Daishan county, Zhoushan, Zhejiang,  China | 30.27 | 122.21 | Su gui-fu, 1988, Plant ash treatment to control Onion fly, Journal of Changjiang Vegetables, 4:29-30 |
| Dalate ,  Mongolia, China | 40.39 | 110.04 | Wang zhuo, Zhou jing-wu, 1965, A preliminary study of Garlic fly, Eumerus strigatus fallen, Acta Entomologica Sinica, 14(2):207-208 |
| Dangyang county, Hubei,China | 30.82 | 111.79 | Zheng lu, Li guo-jing, Guan yiin-chi, Liu xu-hong, Yi xin-grong, Huang jun-bin, 2008, Bionomic and Control of *Delia antiqua* (Meigen) in Garlic, Hubei Agriculture Sciences, 47(8): 917-919 |
| Dastjerdeh, Zanjan, Iran | 36.85 | 48.94 | Samad Khaghaninia, Reze Farshbaf Pourabad, and Davoud Mohammadi, 2009, Control of garlic fly, *Delia antiqua* (Diptera:Anthomyiidae) autumn generation by means of seed coating pesticide, Munis Entomology & Zoology Journal, 4(2):493-497 |
| Datong county,  Qinghai, China | 36.93 | 101.69 | Wu shu-xin, Wang qi-ming, Su you-tai, Yang li-cheng, 2001, Comprehensive prevetion technology of garlic field, Qinghai Agro-Technical extension, 2:63 |
| Datong, Shanxi,  China | 40.06 | 113.25 | Wang zhuo, Zhou jing-wu, 1965, A preliminary study of Garlic fly, Eumerus strigatus fallen, Acta Entomologica Sinica, 14(2):207-208 |
| Datong county, Qinghai, China | 36.90 | 101.70 | Qian qun, Zhang ya-dong, 2004, Cultivation techniques of *Allium fistulosum L.var.giganteum Makion*, Science and Technology of Qinghai Agriculture and Forestry, 2:55-57 |
| Dongling county, Shenyang, Liaoning, China | 41.73 | 123.51 | Teng xue-qiang, 1988, Traps of onion maggot, *Delia antiqua*, Liaoning Agricultural Sciences, 1:33-34 |
| East Vineland, Buena Vista Township, New Jersey, United States | 39.47 | -74.91 | Wesley Kline, Scott Haag, Gerald Ghidiu, 2000, A Comparative Study of Onion Maggot "*Delia Antiqua*" Monitoring Techniques, ASHS Northeast Region Annual Meeting |
| Elba, New York, United States | 43.07 | -78.18 | Brian A.Nault., Benjamin P.Werling, Richard W.Straub, Jan P. Nyrop, 2011, Delaying Onion Planting to Control Onion Maggot (Diptera: Anthomyiidae): Efficacy and Underlying Mechanisms, Journal of Economic Entomology, 104(5):1622-1632 |
| Faku county,  Shenyang,  Liaoning, China | 42.50 | 123.41 | Teng xue-qiang, He zhen-chang, 1989, Observation and research on biological characteristics of Onion maggot, Journal of Shenyang Agricultural University, 20(2):89-94 |
| Fan town, Taian, Shandong, China | 36.20 | 117.24 | Zhang qing-chen, Xue ming, 2010, Study on the Occurrence and Insecticide control techniques of *Delia antiqua* (Meigen) in Tectorial Garlic Field, Master's thesis, Shandong Agriculture University |
| Felsham, United Kingdom | 52.16 | 0.85 | http://www.gbif.org/ |
| Feng county, Xuzhou  Jiangsu, China | 34.69 | 116.60 | Jin yun-lan, Wang cai-yun, Shi hui-ying, 2007, Integrated control of onion maggot, Anhui Agriculture Science Bulletin, 13(11):160 |
| Fuhai county, Altay,  Xinjiang, China | 47.10 | 87.49 | Cai yue-qiang, 1984, Phoxim treatments to control onion maggot larvae, Xinjiang Agricultural Science and Technology, 4:18-19 |
| Fuping county,  Weinan, Shaanxi, China | 34.77 | 109.18 | Wang feng-kui, Ju jiang-li, Zhang hao, 1998, Life history and population Dynamics of *Delia antiqua* (Meigen) and *D.Platura* (Meigen) in Garlic in Central Shannxi, Journal of Northwest Sci-Tech University of Agriculture and Forestry(Natural Science Edition), 26(1):55-59 |
| Gem County, Idaho, United States | 43.86 | -116.51 | Thomas M.M., 1993, Ovipositional Patterns and Larval Movement of *Delia antiqua* (Diptera: Anthomyiidae) on Sprouted Bulb and Seedling Onions, Jouranl of Economic Entomology, 86(5):1440-1445 |
| Genessee counties, New York, United States | 43.07 | -83.67 | Brian A.Nault, Benjamin P.Werling, Richard W.Straub, Jan P.Nyrop, 2011, Delaying Onion Planting to Control Onion Maggot (Diptera: Anthomyiidae): Efficacy and Underlying Mechanisms, Journal of Economic Entomology, 104(5):1622-1632 |
| Grant, Newaygo County, Michigan, United States | 43.33 | -85.80 | Spencer J.L., Miller J.R., 2002, Lifetime ovipositional patterns of mated and virgin onion flies, *Delia antiqua* (Diptera: Anthomyiidae), Journal of Insect Physiology, 48:171-180 |
| Great Staughton, United Kingdom | 52.26 | -3.92 | http://www.gbif.org/ |
| Great Witley, Worcestershire, United Kingdom | 52.28 | -2.35 | Ellis S.A., Escatcherd J., 2007, Bean seed fly (*Delia platura, Delia florilega*) and onion fly (*Delia antiqua*) incidence in England and an evaluation of chemical and biological control options, Annals of Applied Biology, 259-267 |
| Guantao, Handan, Hebei, China | 36.54 | 115.30 | Cui chao-wu, Cheng jin-yan, Cui li, Liu yan, 2011, Occurrence characteristics and control measures of Onion maggot, Modern rural science and technology, 20:28-29 |
| Guantao, Handan, Hebei, China | 36.53 | 115.28 | Guantao agriculture plant protection station |
| Guelph, Ontario, Canada | 43.55 | -80.32 | Joseph L.S. , Marco P.C. , James E.K. , James R.M. , 1995, Onion fly, *Delia antiqua*, oviposition and mating as influenced by insect age and dosage of male reproductive tract extract (Diptera: Anthomyiidae), Journal of Insect Behavior, 8(5): 617-635 |
| Haerbin, Heilongjiang, China | 45.73 | 126.49 | Ji jiao-jiao, Li xue, Wang feng-jiao, Jiang xin-mei, 2007, Relation between different sowing times and occurrence of shallot maggot in welsh onion, 38(6):742-745 |
| Hami, Xinjiang, China | 43.18 | 92.69 | Wang yong-wei, Xu ji-ming, Liu xiu-hua, 1990, The occurrence and prevetion of Onion maggot, Xinjiang Farm Research of Science and Technology, 3:15-16 |
| Hartville, Ohio, United States | 40.97 | -81.34 | Erol Y. , Casey W.H. , 2003, Cyromazine seed treatments to control onion maggot, *Delia antiqua*, on green onions, Journal of Economic Entomology, 96(5):1494-1499 |
| Hejing county, Xinjiang, China | 42.89 | 87.02 | Yu hong-li, Cai cai, Yang xiao-qing, Lou ai-ling, Qu tao, 2008, Damage characteristics and Control of Onion maggot, Xinjiang Agricultural science and technology, 5:64-65 |
| Hemmingford, Quebec, Canada | 45.05 | -73.58 | http://www.gbif.org/ |
| Hohhot, Mongolia, China | 40.84 | 111.80 | Yang gui-qing, Liu shu-qing, Du hai-hua, Xing li-zhong, Yang xiu-zhen, 1986, The Occurrence regularity and control of Onion maggot, Journal of Inner Mongolia college of Agriculture & Animal Husbandry, 7(2):162-168 |
| Hulin, Heilongjiang, China | 45.74 | 132.82 | Zhang feng-mei, Yu xue-mei, 2011, A Study on main disease and pest under garlic field, Heilongjiang Agricultural Sciences, 20:76 |
| Huron County, Ohio, United States | 41.12 | -82.64 | Erol Yildirim, Casey W.H. , 2003, Cyromazine seed treatments to control onion maggot, *Delia antiqua*, on green onions, Journal of Economic Entomology, 96(5):1494-1499 |
| Jiamusi, Heilongjiang, China | 46.79 | 130.29 | Xue yong, 1997, Occurrence and control of Onion fly, Rural agriculture science and technology Information, 9:27 |
| Jimsar, Wulumuqi, Xinjiang, China | 43.91 | 89.40 | Zhao sheng-rui, Du yan, 2007, Method for the prevetion and control of *Delia anqitua*, Xinjiang Agriculture science and technology, 5:47-48 |
| Jimsar, Wulumuqi, Xinjiang, China | 44.07 | 89.21 | Xie yu-qing, Mao jun, Zhu jing, Zhang zhi-dong, 2013, Investigation on Main diseases and insect on White Garlic in Jimsar county, Modern Agricultural Science and Technology, 1:115-117 |
| Jinxiang county, Jining, Shandong, China | 35.07 | 116.31 | Chen li-zhen, Liu fen-gju, Wang yu-zhen, 2002, Integrated control of pests under garlic field, Shandong Vegetables, 3:39 |
| Jinzhong, Shanxi, China | 37.68 | 112.72 | Yang you-lan, Zhang huan-lan, 1994, The occurrence regularity and control measure of Onion fly, Bulletin of Agriculture Science and Technology, 8:26 |
| Kaifeng, Henan, China | 34.79 | 114.28 | Wang feng, 2013, Pesticides seed treatment to control Garlic fly, Pesticide Market News, 23:37 |
| Kangping county, Shenyang, Liaoning, China | 42.75 | 123.35 | Teng xue-qiang, He zhen-chang, 1989, Observation and research on biological characteristics of Onion maggot, Journal of Shenyang Agricultural University, 20(2): 89-94 |
| Kirkwall, Orkney, United Kingdom | 59.02 | -2.96 | http://www.gbif.org/ |
| Kobe, Hyogo, Japan | 34.69 | 135.20 | http://www.gbif.org/ |
| Kodiak, Alaska, United States | 55.96 | -159.35 | http://www.gbif.org/ |
| La Minière, Guyancourt, France | 48.76 | 2.10 | Poprawski T.J., Robert P.H., Maniania N.K., 1985, Susceptibility of the onion maggot, *delia antiqua* (diptera: anthomyiidae), to the mycotoxin destruxin e, The canadian entomologist, 801-802 |
| Le Rheu, France | 48.10 | -1.04 | Biron D.G., Coderre D., Fournet S., Nenon J.P., J.Le lannic, Boivin G., 2005, Larval respiratory systems of two anthomyiid flies, *Delia radicum* and *Delia antiqua* (Diptera: Anthomyiidae), Entomological Society of Canada, 137:163-168 |
| Linyao, Dingxi, Gansu, China | 35.37 | 103.86 | Ma hui-ping, Pan tao, 2011, Occurrence regularity and control of Onion maggot, Agricultural science and technology, 13: 31-32 |
| Liujiang, Liuzhou, Guangxi, China | 24.26 | 109.30 | Wang yu-gui, 2010, Damage Characteristics and innocuous plant protection techniques of Onion maggot, Jilin Vegetable, 5: 63-64 |
| Llangennith, Llanmadoc and Cheriton, United Kingdom | 51.60 | -4.29 | http://www.gbif.org/ |
| Lochain, United Kingdom | 56.54 | -4.29 | http://www.gbif.org/ |
| London, Ontario, Canada | 43.06 | -81.22 | Mcdonald R.S., and Borden J.H., 1997, Host-finding and upwind anemotaxis by *Delia antiqua* (Diptera: Anthomyiidae) in relation to age, ovarian development, and mating status, Environmental Entomology, 26(3):624-631 |
| Mainz, Rheinland Pfalz, Germany | 49.98 | 8.22 | Otto M., Hommes M., 2000, Development of a simulation model for the population dynamics of the onion fly *Delia antiqua* in Germany, EPPO conference on Warning Services for plant protection, 30:115-119 |
| Malheur County, Oregon, United States | 43.71 | -117.06 | Thomas M.M., 1993, Ovipositional Patterns and Larval Movement of *Delia antiqua* (Diptera: Anthomyiidae) on Sprouted Bulb and Seedling Onions, Jouranl of Economic Entomology, 86(5):1440-1445 |
| Mei county, Shaanxi, China | 34.27 | 107.74 | Zhang bin-rang, Zheng duo-mei, 2008, Non-pollution plant protection techniques of Onion maggot in garlic field, Shaanxi Journal of Agricultural Sciences, 6:182-183 |
| Methwold Hythe, Norfolk, United Kingdom | 52.52 | 0.52 | Ellis S.A., Escatcherd J., 2007, Bean seed fly (*Delia platura, Delia florilega*) and onion fly (*Delia antiqua*) incidence in England and an evaluation of chemical and biological control options, Annals of Applied Biology, 259-267 |
| Minle county, Zhangye, Gansu, China | 38.43 | 100.81 | Ge zhen, 2000, Prevention and control of Onion maggot in Minle county, Information of agriculture science and technology, 2: 24 |
| Monheim, Germany | 51.08 | 6.89 | Jorg Romeis, Dirk Ebbinghaus, Jurgen Scherkenbeck, 2003, Factors accounting for the variability in the behavioral response of the onion fly (*Delia antiqua*) to n-dipropyl disulfide, Journal of Chemical Ecology, 29:2131-2142 |
| Montreal, Quebec, Canada | 45.56 | -73.65 | http://www.gbif.org/ |
| Murmansk, Murmanskaya oblast, Russian | 67.88 | 34.91 | http://www.agroatlas.ru/en/content/pests/Delia_antiqua |
| Nanning, Guangxi, China | 22.87 | 108.26 | Du nai-song, 2009, Plant ash treatment to control Onion maggot, Friends of the farmhouse, 9:21 |
| Nanzhao county, Nanyang, Henan, China | 33.49 | 112.43 | Wang ping, 2013, The occurrence of maggot and its prevetion (www.farmers.org.cn) |
| Oswego, New York, United States | 43.44 | -76.46 | Brian A.Nault., Benjamin P.Werling, Richard W.Straub, Jan P. Nyrop, 2011, Delaying Onion Planting to Control Onion Maggot (Diptera: Anthomyiidae): Efficacy and Underlying Mechanisms, Journal of Economic Entomology, 104(5):1622-1632 |
| Owyhee County, Idaho, United States | 42.88 | -115.87 | Thomas M.M., 1993, Ovipositional Patterns and Larval Movement of *Delia antiqua* (Diptera: Anthomyiidae) on Sprouted Bulb and Seedling Onions, Jouranl of Economic Entomology, 86(5):1440-1445 |
| Papenburg, Niedersachsen, Germany | 53.06 | 7.39 | Otto M., Hommes M., 2000, Development of a simulation model for the population dynamics of the onion fly *Delia antiqua* in Germany, EPPO conference on Warning Services for plant protection, 30:115-119 |
| Payette County, Idaho, United States | 44.08 | -116.92 | Thomas M.M., 1993, Ovipositional Patterns and Larval Movement of *Delia antiqua* (Diptera: Anthomyiidae) on Sprouted Bulb and Seedling Onions, Jouranl of Economic Entomology, 86(5):1440-1445 |
| Pine Island, Orange County, New York, United States | 41.29 | -74.45 | Brian A. Nault., Benjamin P.Werling, Richard W.Straub, Jan P.Nyrop, 2011, Delaying Onion Planting to Control Onion Maggot (Diptera: Anthomyiidae): Efficacy and Underlying Mechanisms, Journal of Economic Entomology, 104(5):1622-1632 |
| Pizhou, Jiangsu, China | 34.34 | 118.01 | Liu guang-qin, Mao cheng-xi, Sha guo-dong, Feng wei-min, Lu yu-yu, 2008, Occurrence regularity and comprehensive control technology of onion maggot in Xuzhou, Jiangsu province, Jiangsu Agriculture sciences, 5:110-111 |
| Porthcawl, United Kingdom | 51.46 | -3.66 | http://www.gbif.org/ |
| Potter, New York, United States | 42.70 | -77.22 | Brian A.Nault., Benjamin P.Werling, Richard W.Straub, Jan P. Nyrop, 2011, Delaying Onion Planting to Control Onion Maggot (Diptera: Anthomyiidae): Efficacy and Underlying Mechanisms, Journal of Economic Entomology, 104(5):1622-1632 |
| Qi county, Kaifeng, Henan, China | 34.55 | 114.79 | Luo jin-rong, 1996, The occurrence and control measure of Onion maggot, Agriculture of Henan, 1:13 |
| Qingzhou, Shandong, China | 36.71 | 118.49 | Anqiu county Agriculture Information network (www.aqny.gov.cn) Qingzhou agriculture plant protection station |
| Qishan, Baoji, Shaanxi, China | 34.32 | 107.60 | Wang feng-kui, Ju jiang-li, Zhang hao, 1998, Life history and population Dynamics of *Delia antiqua*(Meigen) and *D.Platura* (Meigen) in Garlic in Central Shannxi, Journal of Northwest Sci-Tech University of Agriculture and Forestry(Natural Science Edition), 26(1):55-59 |
| Riuhta, Hamina, Finland | 60.61 | 27.13 | http://www.gbif.org/ |
| Sainte-Clotilde-de-Beauce, Quebec, Canada | 46.11 | -74.99 | Poprawski T.J., Robert P.H., Majchrowicz I., Boivin G., 1985, Susceptibility of *Delia antiqua* (Diptera: Anthomyiidae) to Eleven Isolates of Entomopathogenic Hyphomycetes, Environmental Entomology, 14:557-561 |
| Sapporo, Hokkaido, Japan | 43.03 | 141.55 | Kazuhiro Tanaka, Tasuhiko Watari, 2011, The onion fly modulates the adult eclosion time in response to amplitude of temperature cycle, Naturwissenschaften, 98:711-715 |
| Sara county, Baotou, Mongolia, China | 40.56 | 110.54 | Wang zhuo, Zhou jing-wu, 1965, A preliminary study of Garlic fly, Eumerus strigatus fallen, Acta Entomologica Sinica, 14(2):207-208 |
| Shenqiu county, Zhoukou, Henan, China | 33.32 | 115.33 | Huang ju-feng, 2013, Disease and Insect Pest Control technology of shallot, *Allium fistulosum*, The Farmers Consultant, 5:45-46 |
| Sheyang, Yangcheng, Jiangsu, China | 33.77 | 120.26 | Li hong-yang, Zhou jia-chun, Zhang jun-xi, Gu hui-ling, 2011, Studies on Occurrence Characteristics of root vermicule in Garlic in Yancheng district and its non-pullution control technology, Acta Agriculturae Jiangxi, 27(4):143-144 |
| Sloviansk, Donetsk Oblast, Ukraine | 48.86 | 37.54 | http://www.smartgardener.com/; http://www.agroatlas.ru/en/content/pests/Delia_antiqua/ |
| Shuozhou, Shanxi, China | 39.33 | 112.39 | Wang zhuo, Zhou jing-wu, 1965, A preliminary study of Garlic fly, Eumerus strigatus fallen, Acta Entomologica Sinica, 14(2):207-208 |
| Sodus, New York, United States | 43.22 | -77.03 | Brian A.N. Benjamin P.W., Richard W.S., Jan P.N., 2011, Delaying Onion Planting to Control Onion Maggot (Diptera: Anthomyiidae): Efficacy and Underlying Mechanisms, Journal of Economic Entomology, 104(5):1622-1632 |
| St Osyth, Essex, United Kingdom | 51.79 | 1.07 | Ellis S.A., Escatcherd J., 2007, Bean seed fly (*Delia platura, Delia florilega*) and onion fly (*Delia antiqua*) incidence in England and an evaluation of chemical and biological control options, Annals of Applied Biology, 259-267 |
| Stark County, Ohio, United States | 40.86 | -81.25 | Erol Y., Casey W.H., 2003, Cyromazine seed treatments to control onion maggot, *Delia antiqua*, on green onions, Journal of Economic Entomology, 96(5):1494-1499 |
| Ste-Anne-de-Bellevue, Quebec, Canada | 45.43 | -73.94 | http://www.gbif.org/ |
| Seoul, Korea | 37.56 | 126.97 | Cho Hyung Chan, Kim Tae Heung Paik, Jong Cheol, 1992, Hymenopteran parasitoids of the onion maggot, *Delia anqitua* in Korea, Korean Journal of Entomology, 22(3): 209-214 |
| Suiling county, Suihua, Heilongjiang, China | 47.23 | 127.11 | Ding yue-zhong, 2005, Control of Onion maggot in garlic and shallot field, Science and Technology of Tianjin Agriculture and Forestry, 6:12-13 |
| Taipusi, Mongolia, China | 41.87 | 115.29 | Wang zhuo, Hou jing-wu, 1965, A preliminary study of Garlic fly, Eumerus strigatus fallen, Acta Entomologica Sinica, 14(2): 207-208 |
| Trial Grounds, United Kingdom | 51.28 | -0.49 | http://www.gbif.org/ |
| Vilniemi, Hamina, Finland | 60.52 | 27.24 | http://www.gbif.org/ |
| Walton-on-Thames, United Kingdom | 51.37 | -0.35 | http://www.gbif.org/ |
| Washington, Idaho, United States | 44.36 | -116.87 | Thomas M.M., 1993, Ovipositional Patterns and Larval Movement of *Delia antiqua* (Diptera: Anthomyiidae) on Sprouted Bulb and Seedling Onions, Journal of Economic Entomology, 86(5):1440-1445 |
| Wellesbourne, United Kingdom | 52.19 | -1.58 | Hartman T.P.V., Southern D.I., 1995, Genome reorganization from polyteny to polyploidy in the nurse cells found in onion fly (*Delia antiqua*) and cabbage root fly (*Delia radicum*) ovaries (Diptera, Anthomyiidae), Chromosome Research, 3:271-280 |
| Wisley, United Kingdom | 51.32 | -0.47 | http://www.gbif.org/ |
| Wisley, United Kingdom | 51.30 | -0.37 | http://www.gbif.org/ |
| Wugong, Xianyang, Shaanxi, China | 34.26 | 108.19 | Wang feng-kui, Ju jiang-li, Zhang hao, 1998, Life history and population Dynamics of *Delia antiqua*(Meigen) and *D.Platura* (Meigen) in Garlic in Central Shannxi, Journal of Northwest Sci-Tech University of Agriculture and Forestry(Natural Science Edition), 26(1):55-59 |
| Wuyuan, Mongolia, China | 41.08 | 108.25 | Wang zhuo, Zhou jing-wu, 1965, A preliminary study of Garlic fly, Eumerus strigatus fallen, Acta Entomologica Sinica, 14(2):207-208 |
| Xiangfan, Hubei, China | 32.01 | 112.18 | Qu hong-jie, Zhao jin-song, He jia-tao, Bie yun-qing, 2006, Occurrence Rule and Control measure of Main diseases and insect pests on *Liriope Spicata (Thunb) Lour*, Hubei Agriculture Sciences, 45(3):337-338 |
| Xiaogan, Hubei, China | 30.91 | 113.89 | Yu hong-zhang, 2011, The prevetion of pests under garlic field, Peasant consultant, 21:32 |
| Xichang, Sichuan, China | 27.89 | 102.26 | Shan chen-ghai, 2011, Harm Characteristics and control of Onion maggot in Xichang, Journal of Changjiang Vegetables, 21:48-49 |
| Xingping county, Xianyang, Shaanxi, China | 34.29 | 108.49 | Liu jin, 2005, Plant ash treatment to control Onion maggot, *Delia antiqua*, Northwest Horticulture, 5:45 |
| Xixia, Shandong, China | 37.34 | 120.85 | Teng xue-qiang, He zhen-chang, 1989, Observation and research on biological characteristics of Onion maggot ,Journal of Shenyang Agricultural University, 20(2):89-94 |
| Xuanhua county, Zhangjiakou, Hebei, China | 40.56 | 115.03 | Zhang gui-fang, Cui fen-ge, 1989, The species of maggot in vegetable field and its prevetion, Modern rural science and technology, 6:15 |
| Xun county, Hebi, Henan, China | 35.68 | 114.53 | Chai guan-qing, 1996, Occurrence regularity and control of Onion maggot, The Farmers Consultant, 6:17 |
| Yakeshi, Mongolia, China | 49.30 | 120.68 | Wang zhuo, Zhou jing-wu, 1965, A preliminary study of Garlic fly, Eumerus strigatus fallen, Acta Entomologica Sinica,14(2):207-208 |
| Yangling town, Xianyang, Shaanxi, China | 34.27 | 108.06 | Wang feng-kui, Ju jiang-li, Zhang hao, 1998, Life history and population Dynamics of *Delia antiqua* (Meigen) and *D.Platura* (Meigen) in Garlic in Central Shannxi, Journal of Northwest Sci-Tech University of Agriculture and Forestry (Natural Science Edition), 26(1):55-59 |
| Yanzhou, Shandong, China | 35.55 | 116.78 | Yuan dong-zheng, Wang lian-gen, 2007, Integrated control of pests under garlic field, Northwest Horticulture, 1:32 |
| Yaolin county, Cixi, Zhejiang, China | 30.17 | 121.32 | Wang qin-hai, Hu jun-da, Cen gui-wu, 1997, Occurrence and control of Onion maggot, Shanghai Vegetables, 5:36 |
| Yiyang county, Luoyang, Henan, China | 34.52 | 112.18 | Dai fa, Li xin, Dua nai-ju, Liu chang-ying, Guo dang, 2007, Studies of occurrence regularity and control techniques of onion, Journal of Henan agriculture science, 4:101-116 |
| Yongji county, Jilin, Jilin, China | 43.65 | 126.48 | Zhang shu-qing, Zhou li, 2004, Non-pollution insect-resistant technology of Vegetable plantation, Jilin Agriculture,6:19 |
| Yuanmou county, Chuxiong, Yunnan, China | 25.71 | 101.87 | Yuan mou county Agriculture Information network ( www.ynagri.gov.cn) |
| Yutian county, Tangshan, Hebei, China | 39.90 | 117.74 | Cai ji-hong, 2012, Main diseases and pests of garlic and its control technology, China Agricultural Information, 10:45 |
| Zhangqiu, Shandong, China | 36.71 | 117.53 | Zhuang zhen-dong, 1995, Observation and Prevetion of Onion maggot, *Delia anqitua*, Plant Protection Technology and Extension, 4:16-17 |
| Zoucheng, Shandong, China | 35.42 | 115.01 | Sun ming-hai, 2007, Control of onion fly under garlic field, Guide of Sci-tech Magazine, 3:15 |
