# Supplementary material for: Predicting the current potential and future world wide distribution of the onion maggot, Delia antiqua using maximum entropy ecological niche modeling
Source: PLoS One. 2017 Feb 3;12(2):e0171190. doi: 10.1371/journal.pone.0171190 (PMC5291381; doi:10.1371/journal.pone.0171190)
Supplement: S2 Table — (DOCX) [file pone.0171190.s002.docx]

| 1 | 1 |  |  |  |  |  |  |  |  |  |  |  |  |  |  |  |  |  |  |
| --- | --- | --- | --- | --- | --- | --- | --- | --- | --- | --- | --- | --- | --- | --- | --- | --- | --- | --- | --- |
| 2 | -.206^*^ | 1 |  |  |  |  |  |  |  |  |  |  |  |  |  |  |  |  |  |
| 3 | .223^*^ | .059 | 1 |  |  |  |  |  |  |  |  |  |  |  |  |  |  |  |  |
| 4 | -.390^**^ | .635^**^ | -.694^**^ | 1 |  |  |  |  |  |  |  |  |  |  |  |  |  |  |  |
| 5 | .616^**^ | .507^**^ | -.190^*^ | .412^**^ | 1 |  |  |  |  |  |  |  |  |  |  |  |  |  |  |
| 6 | .769^**^ | -.605^**^ | .512^**^ | -.873^**^ | .029* | 1 |  |  |  |  |  |  |  |  |  |  |  |  |  |
| 7 | -.352^**^ | .789^**^ | -.544^**^ | .973^**^ | .491^**^ | -.857^**^ | 1 |  |  |  |  |  |  |  |  |  |  |  |  |
| 8 | .439^**^ | .084 | -.527^**^ | .451^**^ | .596^**^ | -.091 | .386^**^ | 1 |  |  |  |  |  |  |  |  |  |  |  |
| 9 | .597^**^ | -.268^**^ | .660^**^ | -.770^**^ | .096 | .832^**^ | -.675^**^ | -.413^**^ | 1 |  |  |  |  |  |  |  |  |  |  |
| 10 | .768^**^ | .226^*^ | -.262^**^ | .288^**^ | .936^**^ | .198^*^ | .310^**^ | .753^**^ | .103 | 1 |  |  |  |  |  |  |  |  |  |
| 11 | .850^**^ | -.496^**^ | .530^**^ | -.816^**^ | .154 | .984^**^ | -.778^**^ | .013 | .820^**^ | .319^**^ | 1 |  |  |  |  |  |  |  |  |
| 12 | .381^**^ | -.619^**^ | -.007 | -.449^**^ | -.115 | .528^**^ | -.520^**^ | .096 | .313^**^ | .084 | .496^**^ | 1 |  |  |  |  |  |  |  |
| 13 | .538^**^ | -.205^*^ | -.218^*^ | .026 | .345^**^ | .248^**^ | -.038 | .600^**^ | .037* | .567^**^ | .320^**^ | .569^**^ | 1 |  |  |  |  |  |  |
| 14 | -.018* | -.595^**^ | .110* | -.515^**^ | -.446^**^ | .391^**^ | -.571^**^ | -.334^**^ | .275^**^ | -.366^**^ | .287^**^ | .745^**^ | -.046 | 1 |  |  |  |  |  |
| 15 | .083 | .451^**^ | -.276^**^ | .559^**^ | .423^**^ | -.391^**^ | .559^**^ | .573^**^ | -.410^**^ | .458^**^ | -.274^**^ | -.373^**^ | .493^**^ | -.810^**^ | 1 |  |  |  |  |
| 16 | .547^**^ | -.321^**^ | -.153 | -.104 | .248^**^ | .342^**^ | -.170 | .519^**^ | .119 | .484^**^ | .399^**^ | .717^**^ | .967^**^ | .104 | .342^**^ | 1 |  |  |  |
| 17 | .006 | -.588^**^ | .122 | -.525^**^ | -.420^**^ | .411^**^ | -.575^**^ | -.337^**^ | .298^**^ | -.347^**^ | .308^**^ | .751^**^ | -.051 | .996^**^ | -.827^**^ | .103 | 1 |  |  |
| 18 | .537^**^ | -.238^**^ | -.287^**^ | .053 | .342^**^ | .228^*^ | -.022 | .706^**^ | -.066 | .579^**^ | .302^**^ | .585^**^ | .939^**^ | -.009 | .422^**^ | .932^**^ | -.010 | 1 |  |
| 19 | -.026 | -.539^**^ | .257^**^ | -.580^**^ | -.462^**^ | .425^**^ | -.609^**^ | -.511^**^ | .446^**^ | -.418^**^ | .319^**^ | .713^**^ | -.032 | .908^**^ | -.749^**^ | .127 | .908^**^ | -.116 | 1 |
|  | 1 | 2 | 3 | 4 | 5 | 6 | 7 | 8 | 9 | 10 | 11 | 12 | 13 | 14 | 15 | 16 | 17 | 18 | 19 |

Table S2: Correlation analysis of environmental variables

**: significant at the 0.05 level *: significant at the 0.1 level
